# Teledentistry and one health: a sustainable framework for oral and planetary health

**DOI:** 10.3389/fdmed.2025.1631548

**Published:** 2025-07-16

**Authors:** Arish Naresh, Zac Morse, Silvana Bettiol

**Affiliations:** ^1^ Department of Community Oral Health Services, Health New Zealand Tairāwhiti, Gisborne, New Zealand; ^2^ College of Health and Medicine, University of Tasmania, Hobart, TAS, Australia; ^3^ College of Dentistry, American University of Iraq-Baghdad, Baghdad, Iraq; ^4^ College of Health and Medicine, University of Tasmania, Hobart, TAS, Australia

**Keywords:** health disparities, workforce challenges, prevention, inequities, sustainibility, dental therapist

## Abstract

This community case study documents the integration of One Health approaches in a teledentistry initiative designed to enhance oral health access for children in Tairāwhiti, New Zealand, a region where Māori children experience significantly higher rates of dental decay than non-Māori. Health New Zealand Tairāwhiti established a virtual dental clinic for children aged 0–2 years, providing care through mobile phones and Zoom. The initiative incorporated the Māori concept of “whakawhanauatanga” to build rapport while conducting assessments and delivering preventive guidance. Between March and December 2024, the service delivered 656 appointments, including 114 for patients in rural locations, resulting in a substantial reduction in travel time and associated carbon emissions. The program utilized a family-based enrollment approach that facilitated access for siblings who might otherwise have remained outside the oral health system into care. This model addresses multiple dimensions of planetary health and the One Health framework simultaneously improving clinical access for vulnerable populations, enhancing preventive education, supporting culturally responsive care for Māori families, and reducing environmental impacts through decreased travel. The findings demonstrate how teledentistry, when designed with sustainability principles, can effectively address oral health inequities while contributing to planetary health through reduced carbon footprint.

## 1 Introduction

Aotearoa New Zealand has long provided free public oral health care for children from birth to 18 years of age with School Dental Service since 1921 (1). The service started with School Dental Nurses, but now Dental or Oral Health Therapists provide treatment in this evolved role. In 2006, the service transformed into Community Oral Health Services under the national strategy “Good Oral Health for All; For Life” (2) with a focus on reducing oral health disparities and increasing access throughout New Zealand (3, 4). However, the dental care service was interrupted by the COVID-19 pandemic, significantly delaying routine and preventive care, especially for children and adolescents (5, 6). These interruptions exacerbated oral health inequities, highlighting the need for innovative delivery models (7).

Telemedicine, including teledentistry, emerged as viable options during the pandemic. It accelerated the adoption of remote and digital dental practices, particularly when patient care was constrained by infection control concerns (7). The influence of technological advancements in healthcare created both new approaches and new service systems (8). Teledentistry rapidly gained traction during this time, offering remote consultations and treatment planning that adhered to social distancing requirements. It enabled patients to access immediate dental care remotely with digital technology, providing the functionality of virtual consultations, remote diagnosis, and treatment planning (8). These tools provided two major benefits: protection from COVID-19 and removal of barriers preventing access to care (9, 10). Furthermore, teledentistry aligns with the World Health Organisation's (WHO) “Global strategy and action plan on oral health 2023–2030” that advocates for the inclusion of oral health in national emergency preparedness and response plans (11). When properly resourced, teledentistry can ensure the continuity of essential oral health services during health emergencies.

Access to oral healthcare remains a significant challenge in many rural and Indigenous communities across Aotearoa New Zealand. The Tairāwhiti region on the East Coast, which has the country's highest proportion of Māori residents (56%) (12) is particularly affected by oral health disparities, where Māori children in the region experience significantly higher rates of dental decay than their non-Māori counterparts (2), driven in part by the intergenerational impacts of colonisation and ongoing systemic inequities (13, 14). Socioeconomic disparities in Tairāwhiti increased between 2013 and 2018, (15), and a shortage of oral health professionals has compounded these issues. The region faces longstanding challenges due to geographic isolation and, more recently, the effects of natural disasters, most notably Cyclone Gabrielle in early 2023. The cyclone caused extensive damage to infrastructure, displaced families, and disrupted health services, further limiting access to care for already vulnerable populations.

The Tairāwhiti Community Oral Health service prioritises care for children overdue for annual exams or in urgent need due to pain or infection, particularly those at higher risk (16, 17). Dental and Oral Health Therapists mainly provide oral health care, and like other regional services in New Zealand, it faces workforce shortages that have led to treatment delays (18, 19, 20). Similar workforce shortages affect Health New Zealand Taranaki and rural Tasmania, Australia (21, 22). These widespread shortages highlight the need for systemic solutions to ensure timely oral health care in underserved areas.

The Health New Zealand Tairāwhiti Community Oral Health Service launched a pilot virtual dental clinic to address these challenges. This initiative addressed two urgent needs: restoring access to oral healthcare in the aftermath of a natural disaster and tackling persistent inequities in oral health outcomes among vulnerable populations due to structural and logistical barriers. Long travel distances, limited appointment availability, and overburdened healthcare services often prevent timely care, particularly for families with young children. The program focused on children aged 0–2 years, aiming to provide early intervention and reduce travel barriers for whānau (Māori word for family unit) and oral health inequities. It aims to deliver timely assessments and treatment for high-risk children, improving equity in service access.

Beyond closing clinical care gaps, the initiative also contributes to environmental sustainability by reducing travel-related carbon emissions, aligning with the principles of planetary health (23). These frameworks highlight the interconnectedness of human, animal, and environmental health, advocating for integrated, cross-sectoral approaches to address health inequities. The One Health model underscores the necessity for interprofessional collaboration to tackle health challenges in underserved rural populations. Recent WHO reports emphasize the importance of investing in One Health actions to prevent and address common health threats affecting humans, animals, plants, and the environment collectively (24). Moreover, the WHO's One Health Joint Plan of Action (2022–2026) outlines strategic actions to enhance global health security through a unified approach (25).

The virtual dental clinic not only delivers essential oral health care but also promotes environmental sustainability by reducing carbon emissions through decreased travel and eco-conscious medical practice adoption. Advancing health equity and improving access to care for underserved communities relies heavily on the integration of telehealth solutions (26).

This innovative model serves two key purposes: providing clinical treatment and supporting educational outreach and community engagement. Digital platforms empower caregivers with accessible information on preventive oral health, fostering proactive health management beyond the clinic (27). The clinic's educational component also reinforces sustainable healthcare practices by considering the environmental context in which communities live.

By addressing both structural and environmental barriers, this pilot highlights the potential for teledentistry to advance health equity in rural and Indigenous communities. The initiative implements an innovative teledental model designed for early intervention in children aged 0–2 years, with a focus on the Māori population in Tairāwhiti; a community disproportionately affected by oral health disparities and further challenged by the aftermath of Cyclone Gabrielle. By improving access, prioritising prevention, and supporting caregiver education, the program aims to overcome challenges such as damaged infrastructure, high travel costs, and geographic isolation—ultimately improving oral health outcomes and reducing inequities across the region.

## 2 Methods

This community case study documents a teledental pilot project initiated by the Tairawhiti Community Oral Health Services. The initiative aimed to improve access to oral healthcare for children aged 0–2 years in Tairāwhiti by offering virtual consultations via mobile phones or the Zoom platform (28). Dental and Oral Health Therapists performed virtual clinical assessments and provided oral healthcare advice via video call, helping to bridge access gaps until face-to-face appointments could be arranged.

Public Health Nurses from the Well Child teams across the region enrolled children into Oral Health Services by completing and forwarding referral forms to the Health New Zealand Tairāwhiti Community Oral Health Service. This enrolment is primarily completed alongside the child's 5 month “Well Child/Tamariki Ora” check, a program that is in place across New Zealand as part of the child's regular health assessment. The target group included children between 0 and 2 years of age who had not previously engaged with oral health services.

The Oral Health Service Coordinator and the lead dental assistant responsible for the virtual clinic initiative coordinated service delivery at a non-clinical level. They contacted parents and caregivers using mobile phones or Zoom to offer flexible appointment options, including mornings, afternoons, evenings, weekends, and school holidays, to accommodate varying family schedules. Due to growing demand, the service expanded from weekdays to 7 days a week.

Appointment times of 15 min were allocated per child, and the clinician called within the agreed-upon time window to conduct the remote assessment and provide oral health guidance. During the first part of the appointment, the clinician focused on building rapport through the concept of “*whakawhanauatanga*” (the concept in Māori culture that is about getting to know the other party), which emphasises the importance of getting to know each other and fostering a sense of connection (29). This approach facilitated open dialogue about the family's social history, as well as the health enablers and barriers they may face. The clinician began by gently asking questions to better understand the child's oral health and overall well-being.

These questions included:

1. Is your child experiencing any toothache?
2. Is your child having any issues with chewing their food?
3. Have you noticed any holes in your child's teeth or ulcers on their gums?
4. Has the child suffered any injuries to their teeth, gums, or lips in the past 6 months?
5. Describe the child's toothbrushing pattern and the type of toothpaste being used.
6. Describe the diet that your child has, e.g., do they have lollies and chocolates frequently?
7. Does your child ever wake up in the middle of the night complaining of toothache?
8. Do you have any other queries or concerns?
9. Are there any cultural practices or beliefs that influence your child's oral health care?
10. What support systems do you have in place for managing your child's health and dental care?

These questions were not asked in order, but the conversation was focused on parental engagement, and the questions were covered as part of the overall conversation to ensure the whole engagement was more natural and put the parent at ease.

These questions not only addressed the child's dental health but also allowed the clinician to gain insights into the family's context and support systems. The clinician would then verbally provide the parent or caregiver with information about the oral health service and how to reach the services should the parent/caregiver have any concerns. The clinician also advised them on what to look out for in terms of dental abscesses, dental-related injuries, and the importance of the New Zealand government's insurance for accidental injuries [the Accident Compensation Cooperation (ACC)] registration for dental injuries.

Verbal informed consent was obtained from the primary caregiver/parent, and if the parent preferred not to have a teledental consult, their details were passed on to the administrative team to schedule an in-person appointment to ensure the caregiver/parent always had a choice and ability to opt out of the program. Basic operational technologies, including Excel and Word documents, allow data collection as part of the current implementation. The data was collected using phone and Zoom calls and then recorded electronically using Excel and Word files. The project focussed on 0 to 2-year-olds who are deemed low risk and hence suited for teledentistry. The data was recorded using a laptop issued by the public health services with password protection, multi factor authentication mechanism in place and the data is securely stored on Health New Zealand Tairawhiti's secure drives. The organisation is part of New Zealand's Ministry of Health and abides by HL7 protocols as well as the Privacy Act 2020 and Public Records Act 2005.

## 3 Results

### 3.1 Project output

Between March 2024 and December 2024, 656 appointments were delivered through teledentistry**.** The pilot project resulted in 59 patients requiring follow-up care and 47 additional siblings being enrolled in the program, with 114 appointments provided to patients in rural and remote locations. This reduced driving time by approximately 456 h for these communities. The 456-h reduction in driving time was calculated based on average return journeys from the service's fixed oral health facility in Gisborne City to rural communities, including Te Karaka in the western region and Ruatoria near the East Cape of the Tairāwhiti district.

## 4 Parental engagement and satisfaction

The parents who participated in the teledental consultations expressed strong satisfaction with the service because it provided efficient and accessible dental care. Parents perception of the project was that they appreciated that the teleconsultation service operated 7 days a week, allowing them to schedule appointments at times convenient for their busy routines. The program's family-based enrolment approach benefited households with multiple children, enabling parents to register siblings of various ages who might otherwise have remained outside the oral health system. The program allowed patients to request appointments that included in-person and teledental services according to their necessities.

Eligible parents expressed appreciation during the consultations, having reduced travel obligations because most necessary dental guidance could reach their children through the teledental program. Simplified access became a vital benefit, relieving families from transportation struggles that affected many in rural and remote regions. If parents raised concerns about their child's oral health condition or dental concerns, they received a follow-up appointment for in-person care. Overall, the teledental initiative improved access to care and supported a shift toward proactive and family-centred oral health management using technology as an enabler. The pilot project did not evaluate clinical outcomes in detail and this is an area that can be further evaluated in future research.

## 5 Discussion

The teledentistry Pilot Project developed by Health New Zealand Tairāwhiti makes significant progress toward enhancing parental participation and oral healthcare access for families from disadvantaged economic backgrounds. The use of teledental services enables families to avoid time-consuming travel to Gisborne City, which previously created financial and logistical barriers for many families. Rural families encounter additional difficulties because their dental service locations surpass 100 km in the Tairāwhiti region, and their transportation costs, along with work-time expenses, add to the burden (30). A systemic review and meta-analysis in Australia determined that teledentistry utilisation supports that teledentistry increases healthcare access, especially for people in regional, rural and remote areas. It affirms teledentistry as an effective screening tool for caries (31).

Virtual consultation delivery through this project helps reduce family obstacles to parental involvement in their children's oral health care. Teledental appointments provide the opportunity to discuss dietary habits, toothbrushing habits, and any concerns such as dental injuries. They also provide a platform to monitor minor oral health issues in pediatric patients, such as neonatal or natal teeth or eruption cysts (8). A review of six systematic reviews and 817 citations that included over 7,000 patients supported teledentistry as an effective means for dental referral, treatment planning, and reinforcing oral health compliance (32). Early adopters of teledentistry found the transition much easier during COVID-19; had better success in supporting their patients during the pandemic period and see teledentistry playing a valuable role in the long-term future (31).

The teledental service enables parents to schedule appointments on any day of the week because it operates without restrictions, thereby accommodating their busy routines. Adaptable appointment scheduling has led to better parent involvement since parents prefer dental care participation when times are convenient for them (32). Parents can now use the initiative to enroll their younger children or older children who lack system access, which allows them to receive dental consultations specific to their needs. The comprehensive approach covers both urgent dental treatment needs and creates a family environment that improves health management practices, which are essential to address excessive dental decay reported in Tairāwhiti region children (3).

Basic operational technologies, including Excel and Word documents, allow data collection as part of the current implementation. Direct data entry into Titanium (national dental patient management system) represents the most appropriate way forward because it is the digital dental record system for the region. Implementing this system would create data management efficiency along with improved record accuracy to produce better operational efficiency in healthcare provider workflows. Titanium Dental Solutions is a widely used dental patient software system in New Zealand and Australia, enabling the secure recording of patient information, charting, radiographs, and other key information. The data can then also be downloaded and passed on to patients at their request or the request of others, such as the coroner, with relevant approvals.

Digital solutions within healthcare systems have risen in importance because they help healthcare operations become efficient and enhance patient treatment results (33). An extensive clinical governance framework must be developed in parallel with the 2-to-3-year duration of the pilot program. A developed framework will collect every finding while creating expansion guidelines for the program implementation. Other primary care providers should engage in collaboration because of the shortage of oral health clinicians in current practice. Various health professionals working together in transdisciplinary teams will help enhance the effectiveness of oral health care delivery for children. The New Zealand healthcare system already operates “Lift the Lip” together with “B4School Checks,” which enables children aged 0–2 years to receive early access to oral health services. The program could solve current oral health service capacity issues by incorporating comprehensive oral health screening into existing Child Health programs that can be delivered through telehealth/teledental approach to increase access for children through a transdisciplinary approach that involves utilisation of professions such as nurses and general practitioners.

A well-developed teledental toolkit that outlines how the transdisciplinary practice can be implemented would enhance oral health outcomes and reduce disparities in dental service distribution. Public health services require a better connection between their separate functions since dental care frequently receives insufficient attention, which highlights the requirement to integrate healthcare approaches that focus on children's complete well-being. According to Foláyan (34), handling the intergenerational colonial legacies experienced by Māori communities is necessary to enhance their health results. Organisations should use a team approach to integrate dental health programs with core healthcare systems while establishing an equal framework for better health system sustainability.

Operationalising sustainable oral health models, such as the teledentistry approaches described in this community case study, would encourage collaborative co-design with Māori communities, healthcare providers, and policymakers. This participatory approach enables the development of culturally responsive interventions that simultaneously improve oral health access, reduce inequities, utilize appropriate technologies, and contribute to planetary health through reduced carbon emissions (35).

The teledentistry pilot contributes significantly to planetary health goals by reducing travel-generated carbon emissions from patient transportation. This environmental benefit aligns with broader sustainability principles in healthcare delivery, as described by Hackley and Luca (36), who noted that prevention-focused oral care produces substantially lower environmental impacts compared to treatment-focused approaches through reduced material consumption, waste generation, and carbon emissions. By integrating both environmental sustainability and improved healthcare access, the teledentistry model exemplifies the One Health approach, uniting ecological and human well-being while simultaneously addressing oral health inequities among rural and Māori populations. Future teledentistry implementations should systematically measure these environmental impacts to further quantify the sustainability benefits of virtual dental care delivery (37).

## 6 Conclusion

The teledentistry Pilot Project serves as a progressive model for enhancing access to dental care, particularly for rural and remote communities. By significantly increasing patient engagement and reducing inequalities, it effectively addresses the oral health needs of vulnerable populations, including Māori whānau in geographically isolated regions such as Tairāwhiti. The project empowers parents with greater flexibility in managing their children's dental health but also aligns with environmental sustainability through its technology-driven approach. To further advance these efforts and tackle oral health inequities among preschool-aged children, it is recommended that a national teledental toolkit be developed. This initiative would build on the project's successes and provide a scalable solution to improve oral health outcomes across communities in Aotearoa New Zealand. While particularly beneficial for rural and regional communities in places like North and Northwest Tasmania with similar workforce shortages, the model has potential applications in diverse global settings where geographic isolation, limited oral health workforce, and health inequities create barriers to care. The core principles of this approach, i.e., early intervention, reduced travel burdens, cultural responsiveness, and environmental sustainability, offer a framework that could be adapted to various healthcare systems worldwide, particularly those serving indigenous and rural populations. This pilot project demonstrates how teledentistry, when integrated with One Health principles, can deliver improved oral health outcomes to vulnerable populations through enhanced rural healthcare delivery. To maximize its global impact, future efforts should include developing specific strategies and adaptable frameworks that consider diverse cultural and healthcare contexts, thereby making the model more universally applicable. This approach requires buy-in from policymakers and stakeholders for scaling up and must be supported by a framework ensuring clinical governance, corporate governance, and cultural responsiveness.

## Data Availability

The original contributions presented in the study are included in the article/Supplementary Material, further inquiries can be directed to the corresponding author.
